# Bubbles Quantified *In vivo* by Ultrasound Relates to Amount of Gas Detected *Post-mortem* in Rabbits Decompressed from High Pressure

**DOI:** 10.3389/fphys.2016.00310

**Published:** 2016-07-21

**Authors:** Yara Bernaldo de Quirós, Andreas Møllerløkken, Marianne B. Havnes, Alf O. Brubakk, Oscar González-Díaz, Antonio Fernández

**Affiliations:** ^1^Veterinary Histology and Pathology, Department of Morphology, Veterinary School, Institute of Animal Health, University of Las Palmas de Gran CanariaLas Palmas, Spain; ^2^Department of Circulation and Medical Imaging, Norwegian University of Science and TechnologyTrondheim, Norway; ^3^Physical and Chemical Instrumental Center for the Development of Applied Research Technology and Scientific Estate, University of Las Palmas de Gran CanariaLas Palmas, Spain

**Keywords:** decompression sickness, blockage of circulation, bubble grade, gas bubbles, gas emboli

## Abstract

The pathophysiological mechanism of decompression sickness is not fully understood but there is evidence that it can be caused by intravascular and autochthonous bubbles. Doppler ultrasound at a given circulatory location is used to detect and quantify the presence of intravascular gas bubbles as an indicator of decompression stress. In this manuscript we studied the relationship between presence and quantity of gas bubbles by echosonography of the pulmonary artery of anesthetized, air-breathing New Zealand White rabbits that were compressed and decompressed. Mortality rate, presence, quantity, and distribution of gas bubbles elsewhere in the body was examined postmortem. We found a strong positive relationship between high ultrasound bubble grades in the pulmonary artery, sudden death, and high amount of intra and extra vascular gas bubbles widespread throughout the entire organism. In contrast, animals with lower bubble grades survived for 1 h after decompression until sacrificed, and showed no gas bubbles during dissection.

## Introduction

Decompression illness is a term used to describe diseases caused by intravascular or extravascular bubbles that are formed as a result of reduction in environmental pressure (decompression). The term covers both arterial gas embolism and decompression sickness (DCS; Vann et al., 2011). Bubbles can have mechanical, embolic, and biochemical effects with manifestations ranging from trivial to fatal (Vann et al., 2011).

Bert noted that the presence of gas bubbles in the blood was not a necessary cause of death or even visible symptoms (Bert, 1878). However, he suggested that gas in the vascular system or in the tissues must cause pain and decompression symptoms. He further suggested that the risks of paralysis or death depends upon the size of the gas bubble and hence, the capability to block the circulation.

The presence and amount of intravascular circulating bubbles can be determined by ultrasound technology. This technology is used in scuba divers as a non-invasive system for detection of intravascular bubbles after diving schedules, as an indicator of decompression stress (Doolette and Mitchell, 2001; Pollock, 2007; Germonpre et al., 2014; Møllerløkken et al., 2016). The absence of detectable bubbles has been found to highly correlate with the absence of DCS symptoms (Sawatzky, 1991), and large quantities of intravascular bubbles predispose for the development of DCS (Evans et al., 1972; Neuman et al., 1976; Spencer, 1976; Gardette, 1979; Sawatzky, 1991). However, the relationship between intravascular bubbles detected by ultrasound and DCS is largely probabilistic, with no absolute threshold for number or size of bubbles below which there is no risk (Weathersby et al., 1984). DCS has occurred in divers with few bubbles detected by Doppler (Bayne et al., 1985). In contrast, no symptoms of DCS were recorded in individuals with large quantities of intravascular bubbles (Nishi et al., 2003). A recent study has shown that venous gas emboli detected by two-dimensional echocardiography are an imperfect surrogate endpoint for DCS (Doolette, 2016). There are no studies on detectable bubbles and lethal decompression in human controlled studies for obvious ethical reasons; hence the relationship between detectable bubbles by ultrasound and fatal decompression remains unknown.

The relationship between intravascular gas bubbles in a given localization determined by ultrasound and the amount of gas bubbles and the distribution of these intravascularly or extravascularly elsewhere in the organism is unknown. This relationship might contribute to understand better the pathophysiology of DCS. In the present study, we compared the presence and amount of intravascular circulatory gas bubbles at the pulmonary artery of New Zealand White (NZW) rabbits after decompression using ultrasound, to the presence and amount of gas bubbles in different vascular and extravascular body locations found grossly *post mortem* (PM) during necropsy.

## Materials and methods

### Animals

Male NZW rabbits (4 from Animal Supply Center of the Negrin Hospital, Spain, and 14 from the Unit of Comparative Medicine, St Olav University Hospital NTNU, Norway) of 2.5–3.8 kg weigh were used in this experiment. All experiments were conducted in accordance with the European Union regulations for laboratory animals. Experimental protocols for the control and putrefaction studies were performed in Spain and approved by the Ethical Committee for Animal Experiments of the University of Las Palmas de Gran Canaria (Spain). The Norwegian Committee for Animal Experiments approved the protocol for the compression/decompression study where it was carried out.

### Experimental protocol

Animals were assigned into the following two experimental groups (a) control or gas putrefaction studies (*n* = 4), and (b) compression/decompression treatment (*n* = 14). All experiments were conducted under surgical anesthesia (Medetomidine (0.5 mg·kg^−1^) and Ketamine (25 mg·kg^−1^) subcutaneously).

(a) **Control Group**
  After anesthesia animals were euthanized with an intraperitoneal injection of pentobarbital (200 mg·kg^−1^). Dead animals were kept in hermetically sealed plastic boxes for biological material at room temperature (24.0 ± 0.8°C) for 1, 3, 6, and 12 h PM (*n* = 1 for each time) before necropsies were performed.
(b) **Compression/Decompression Model**
  Anesthetized NZW rabbits were compressed in pairs, in a dry hyperbaric chamber (Animal Chamber System, NUT, Haugesund, Norway) to 8 atmospheres absolute (ATA) with 45 min bottom time followed by a fast decompression (0.33 m/s). During the dive, animals breathed compressed air (Medical air, Yara Praxair, Oslo, Norway). The dive profile was planned to be aggressive. Previous diving experiments in rabbits showed that a dive to 6ATA during 45 min showed a low mortality rate (Shim et al., 1967; Tanoue et al., 1987; Su et al., 2004). After decompression, the pulmonary artery and the aorta of the rabbits were monitored by ultrasound for *in vivo* bubble detection by a 10 MHz transducer connected to an ultrasound scanner (GE Vivid Five, Vingmed Ultrasound AS, Norway). Bubbles were detected as bright spots. The abundance of gas bubbles was evaluated using the Eftedal and Brubakk (EB) grading scale from 0 to 5 (Table 1; Eftedal and Brubakk, 1997). Ultrasound monitoring was repeated every 15 min for 1 h following decompression, or until death of the animal. Animals that survived for 1 h after decompression were euthanized with an intraperitoneal injection of pentobarbital (200 mg·kg^−1^). The bodies were placed in hermetically sealed plastic boxes for biological material at room temperature (23.3 ± 1.3°C) for 0, 20, and 40 min, and 1, 3, 6, and 12 h (*n* = 2 for each time) PM.

**Table 1:** **Grading code for ultrasonic images following Eftedal and Brubakk (1997)**.

**Grade**	**Definition**
0	No observable bubbles
1	Occasional bubble
2	At least 1 bubble every 4 heart cycles
3	At least 1 bubbles every heart cycle
4	At least 1 bubble per cm^2^ in every image
5	“White-out”: single bubbles cannot be discriminated

### *Post-mortem* procedures

Animals were carefully dissected and the presence of gas was evaluated using a standardized gas score index (Bernaldo de Quirós et al., 2016). For this purpose a gas score from 0 to VI was given to different vascular locations (subcutaneous, mesenteric, femoral, and coronary veins as well as the right atrium) and a gas score from 0 to III was used to evaluate the presence of gas beneath the capsule of different tissues (subcapsular emphysema), and within adipose tissues (interstitial emphysema; Tables 2, 3). These scores were used to obtain a new gas score index to represent the global or total gas score for each animal. This new index was obtained by the summation of the gas scores for each tissue, thus total gas score in each animal ranges from 0 to 42 (Table 3). The production of putrefaction gases with PM time was studied in a previous manuscript of this same experiment including animals with longer PM hours (Bernaldo de Quirós et al., 2013, 2016). Results from gas composition analyses and gas score analyses indicated that putrefaction gases were not significant in the animals with 27 h PM or less. Putrefaction gases were not detected in animals with 12 h PM or less. For this study we have included only those animals that were considered fresh and free of putrefaction gases following gas composition and gas scoring analyses (Bernaldo de Quirós et al., 2013, 2016).

**Table 2:** **Definition of gas score index for ***post-mortem*** examinations following Bernaldo de Quirós et al. (2016)**.

**Gas score**	**Definition**
**INTRAVASCULAR**	
0	Absence of bubbles
I	Occasional bubble
II	Few bubbles and/or discontinuities of blood
III	Few bubbles and large discontinuities of blood
IV	Moderate presence of bubbles
V	Abundant presence of bubbles
VI	Complete sections of vessels filled with gas
**EXTRA-VASCULAR**	
0	Absence of gas
I	Scarce presence: affecting only 1 organ
II	Moderate presence of gas: in more than 1 organ
III	Abundant presence of gas: systemic

**Table 3:** **Calculation of total gas score for each animal following Bernaldo de Quirós et al. (2016)**.

**Identification**	**Subcutaneous v**.	**Mesenteric v**.	**Femoral v**.	**Vena cava**	**Right atrium**	**Coronary v**.	**Interstitial emphysema in fatty tissue**	**Subcapsular emphysema**	**Total**
Animal n	0–VI	0–VI	0–VI	0–VI	0–VI	0–VI	0–III	0–III	0–42

### Data analysis

SPSS Statistics 17.0 software was used for statistical analysis. The correlation between EB bubble grade and PM gas scoring was analyzed by Spearman's rank correlation coefficient. PM gas scoring data was partitioned in two groups using K-means clustering. The statistical difference between these two groups was assessed by Mann–Whitney *U*-test. Statistical significance was set at *p* < 0.05.

## Results

### Post-diving observations

A large individual variability in EB bubble grade was found: 2/14 NZW rabbits (14.3%) presented with an EB bubble grade of 0; 2/14 NZW rabbits (14.3%) presented with an EB bubble grade of 1; 1/14 NZW rabbits (7.1%) presented with an EB bubble grade of 2; 1/14 NZW rabbits (7.1%) presented with an EB bubble grade of 3; 4/14 NZW rabbits (28.6%) presented with an EB bubble grade of 4; and 4/14 NZW rabbits (28.6) presented with an EB bubble grade of 5. About half of the animals (42.9%) presented with an EB bubble grade of 0–3 while the other 57.1% of the animals presented with an EB bubble grade of 4 or 5. Animals with bubble grade 0–3 survived for 1 h and were euthanized according to the experimental protocol. In contrast, all the animals with bubble grade 4 or 5 showed severe respiratory distress signs and died within 5 to 35 min after decompression.

### PM examinations

#### Control group

Grossly, there was an absence of intravascular and extravascular gas bubbles. Occasionally single or very few scattered gas bubbles were observed. Minimum and maximum gas score values for this group were 0 and 4, respectively. Mode gas score value was 0.

#### Diving group: EB bubble grade 0–3

All these animals (*n* = 6) survived for 1 h after decompression and were euthanized following the protocol. There was an absence of intravascular and extravascular gas bubbles. Gas score was 0 for all these animals.

#### Diving group: EB bubble grades 4 and 5

**Figure 1:**
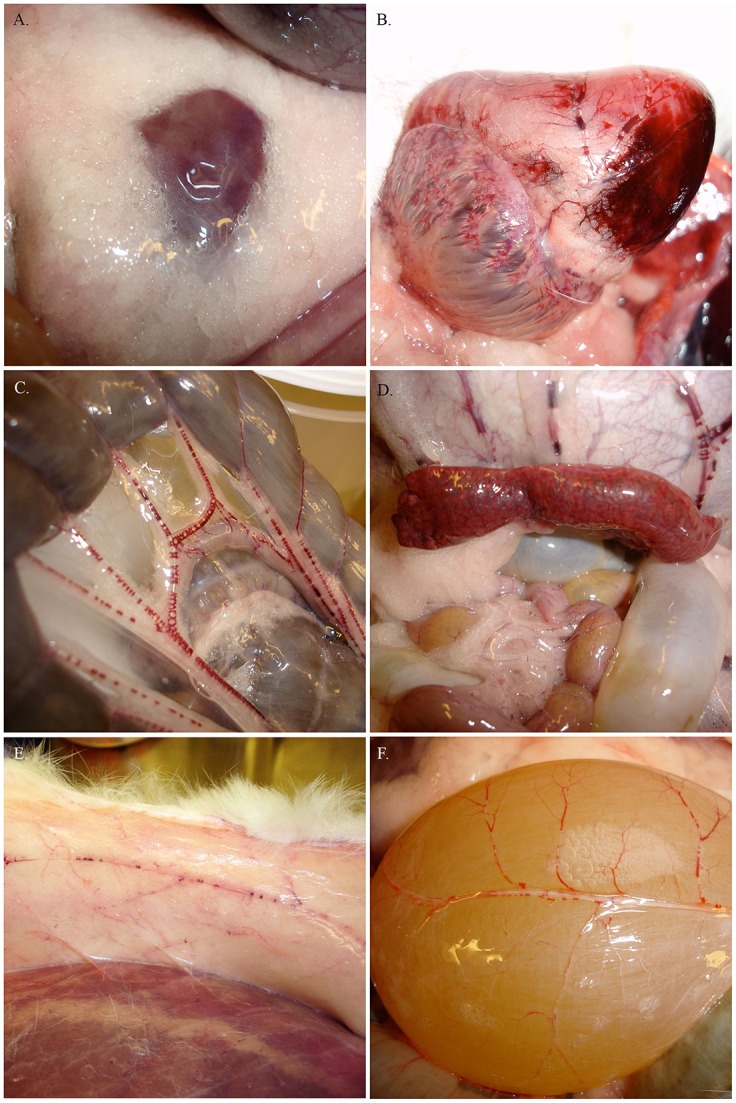
**Pictures from PM examinations of the rabbits with bubble grades 4 and 5 showing gas in the abdominal adipose tissue (A) and extensive gas embolism including right atrium and coronary veins (B), mesenteric veins (C), gastric veins and filling of the spleen (D), subcutaneous veins (E), and urinary bladder veins (F) among other veins**.

These animals (*n* = 8) died within 5–35 min after decompression. Gas was abundant and disseminated in all the venous circulatory system and in the tissues, including peripheral veins and the abdominal adipose tissue. There were veins completely filled with gas showing evident vascular obstruction. In many cases, lesions such as hemorrhages were observed in association with the bubbles (Figure 1). Minimum and maximum gas score values for this group were 29 and 39, respectively. Mode gas score value was 34.

### Relationship between EB bubble grade and PM gas score

**Figure 2:**
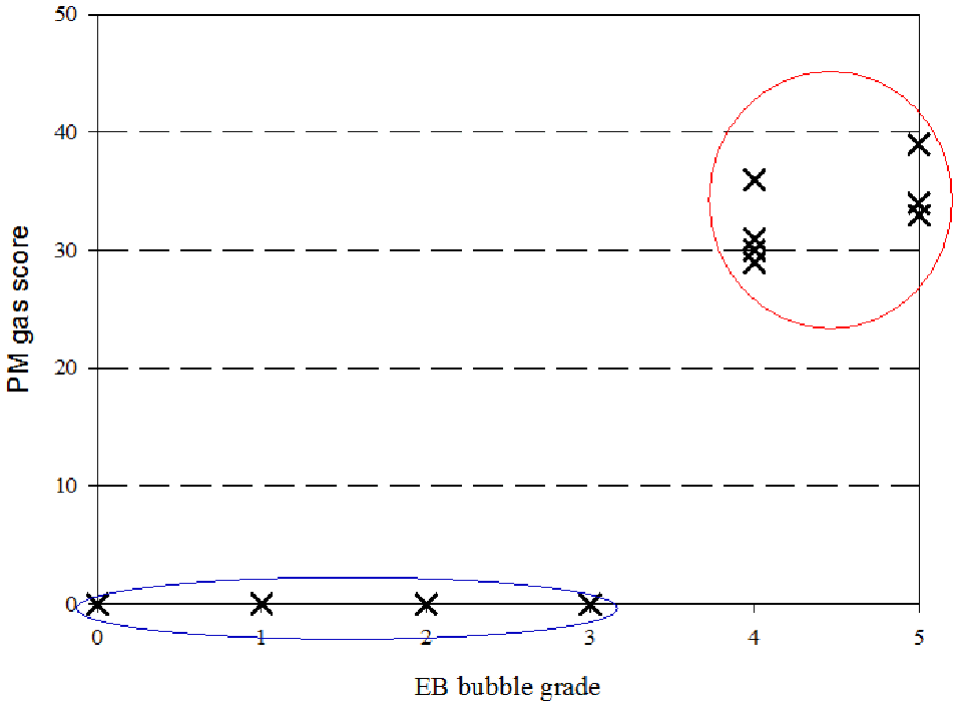
**Correlation between PM gas scoring and ***in vivo*** EB bubble grade**. Two clusters were distinguished following the K-means cluster test: Cluster 1 was composed by animals with gas score of 0 and EB bubble grade of 3 or lower (blue circle), and 2 composed by animals with gas score of 29 or higher and EB bubble grade of 4 or higher (red circle).

The K-means cluster test differentiated two clusters attending to PM gas scoring. Cluster 1 was composed by animals (*n* = 6) with gas score of 0, and center at 0 gas score. Cluster 2 was composed by animals (*n* = 8) with gas score of 29 or higher, and center at 33 gas score. Distances between final cluster centers was 33.3. Cluster 1 and 2 were statistically significant different (*P* < 0.001; Figure 2).

Cluster data was crossed with EB bubble grade. Animals from cluster 1 presented with EB bubble grade of 3 or lower. In contrast, animals from cluster 2 all presented with EB bubble grade of 4 or higher (Figure 2). EB bubble grade and PM gas scoring were found to be strongly correlated (rho = 0.883; *P* ≤ 0.0001).

## Discussion

To the authors knowledge this is the first study describing the relationship between bubble grades detected by ultrasound in the pulmonary artery *in vivo*, and the presence of macroscopic bubbles elsewhere in the organism.

The present study showed a high inter-individual variability in bubble grades detected by ultrasound, despite having similar subjects (species, gender, weight) exposed to the same treatment. This result supports previous work by Eckenhoff et al. (1990) who reported a considerable inter and intra-individual variability in bubble formation at any level of exposure (Eckenhoff et al., 1990).

The main finding from this study was that EB bubble grade measured *in vivo* by ultrasound correlated to mortality and to gas score determined PM: animals with EB bubble grades 1–3 survived for 1 h after decompression until sacrificed according to protocol and presented with low gas score when examined PM, while animals with EB bubble grades 4–5 died within 5–35 min after decompression and presented with high gas score of gas bubbles widely dispersed in different veins, including peripheral veins, and in the adipose abdominal tissue when examined PM.

The mortality result was unexpected since large quantities of intravascular bubbles have been reported in asymptomatic apparently healthy divers (Nishi et al., 2003). PM examination showed only two treatment responses: large amount of gas bubbles within veins and tissues in animals dying shortly after decompression vs. absence or very few gas bubbles in animals which survived and were later sacrificed. Similar observations on survival and amount of bubbles detected PM have been previously reported in sparrows, mice, rats, pigs, cats, dogs, and rabbits (including NZW), that have been rapidly decompressed from high pressures (Bert, 1878; Eggleton et al., 1945; Lever et al., 1966; Shim et al., 1967). Most of these studies support our findings regardless species and exposure pressure levels. Bert's results were more varied but they also showed that animals dying rapidly after sudden decompression always presented with large amounts of gas bubbles in the venous side of the circulatory system (Bert, 1878).

It is interesting to note that regardless the different sensitivity to decompression (demonstrated by higher prevalence of DCS symptoms and/or morbidity) of different species (Bert, 1878; Eggleton et al., 1945), necropsy findings were similar to our results showing a dual response in dead vs. surviving animals (Eggleton et al., 1945; Shim et al., 1967): all animals dying shortly after decompression showed large amounts of gas bubbles in the veins regardless of animal species, or hyperbaric exposure.

Our animals that died shortly after decompression (EB bubble grades 4 and 5) also presented with gas bubbles within the abdominal adipose tissue. This finding has been described before and used as a symptom to diagnose DCS in rats (Hyldegaard and Madsen, 1989).

The dual response (presence and high amounts of gas bubbles vs. absence of gas bubbles) of the gas score was very different from the high individual variability in EB bubbles grades measured by ultrasound. Differences found between EB bubble grade measured by ultrasound and PM gas score examination could be due to different resolution limits of the two methods. Monitoring with high-resolution ultrasound renders the possibility of detecting smaller bubbles than is possible by visual examination. Additionally, small bubbles are more susceptible to be trapped and excreted in the pulmonary capillaries (Francis and Simon, 2003) or simply get diluted. According to LaPlace equation, smaller bubbles have larger inner pressure, thus dissolve more quickly than large bubbles (Hrncír, 1996). Large bubbles are more stable and easier to see macroscopically. Additional limitation of this method is the low number of cases studied. More studies using fresh animals should be carried out to reassure the results presented in this manuscript.

Future pathological studies comparing animals with EB bubble grades 0–3 and EB bubble grades 4–5 might help us to understand better the unexpected mortality rate described in this study, as well as the pathophysiological mechanism of DCS.

This study has focused in lethal decompression, which is rare among human divers except for the most severe accidents, thus the application of these results in human clinical medicine might be limited. On the other hand, studying diseases in their most extreme expression might be helpful to detect better pathophysiological mechanisms of the disease that might not be detected otherwise. The results from the present experiment should be considered carefully due to the low number of individuals studied, but the results suggest a strong relationship between fatal decompression and high loads of gas emboli in NZW rabbits. This relationship remains unclear in less severe decompression cases in human divers (Evans et al., 1972; Neuman et al., 1976; Spencer, 1976; Gardette, 1979; Bayne et al., 1985; Sawatzky, 1991; Nishi et al., 2003).

Another limitation to our data is that there are to our knowledge no studies available today that describes where decompression induced vascular bubbles do originate from, and whether there are specific tissues or organs that are more “bubble-producers” than others. Recent studies are attempting to detect small stationary bubbles in tissues using Dual-frequency ultrasound (Swan et al., 2011, 2014). In this regard, the total gas score estimated for each animal might underweight the pathophysiological importance of high bubble loads in a single tissue. The gas score for each tissue might be more informative for that respect.

In summary, in the present study we have reported a dual response of presence of gas bubbles in postmortem examinations (high amount of gas bubbles vs. absence of gas bubbles) in animals of the same species, gender, and similar weight, exposed to the same diving profile. *In vivo* ultrasound measurements at the pulmonary artery related to quantity of gas widely distributed in the venous system in decompression cases. High bubble grades were related to high gas score causing death within 35 min after decompression in NZW rabbits.

## Author contributions

AM and MH contributed to the experiments, interpretation of the results and provide their expertise. AB, OG, and AF, supervised the work, contributed to the discussion of the results and provide their respective expertise. YB carried out the experiments, data analyses and interpreted the results. All authors listed contributed to the writing of the manuscript.

### Conflict of interest statement

The authors declare that the research was conducted in the absence of any commercial or financial relationships that could be construed as a potential conflict of interest.
